# Iron-sulfur cluster biogenesis and regulation of intracellular iron homeostasis in *Escherichia coli*

**DOI:** 10.1093/mtomcs/mfaf040

**Published:** 2025-11-20

**Authors:** Huangen Ding

**Affiliations:** Department of Biological Sciences, Louisiana State University, Baton Rouge, LA 70803, USA

## Abstract

Iron is an essential metal for almost all living organisms. The major ‘consumers’ of intracellular iron content are a group of proteins that require an iron-sulfur cluster for their functions. It has been shown that iron-sulfur clusters in proteins are assembled by a set of highly conserved proteins using intracellular free iron and L-cysteine as iron and sulfide sources, respectively. Ironically, excess iron is detrimental to cells as free ferrous iron promotes the production of reactive oxygen species via the Fenton reaction. In *Escherichia coli*, intracellular iron homeostasis is regulated primarily by a global transcription factor Fur (ferric uptake regulator). Since its discovery, it had been assumed that Fur binds ferrous iron to regulate intracellular iron homeostasis, oxidative stress response, and bacterial virulence, among others. However, the proposed ‘iron-bound’ Fur had never been identified in *E. coli* or any other bacteria. Recent studies have revealed that *E. coli* Fur binds a unique [2Fe-2S] cluster in response to elevation of intracellular free iron content, and that the [2Fe-2S] cluster in Fur is enzymatically assembled by the iron-sulfur cluster biogenesis machinery. Because Fur also regulates the expression of the genes encoding the iron-sulfur cluster assembly machinery, Fur represents a key link between biogenesis of iron-sulfur clusters and regulation of intracellular iron homeostasis in bacteria.

## Graphical Abstract

**Graphical Abstract:**
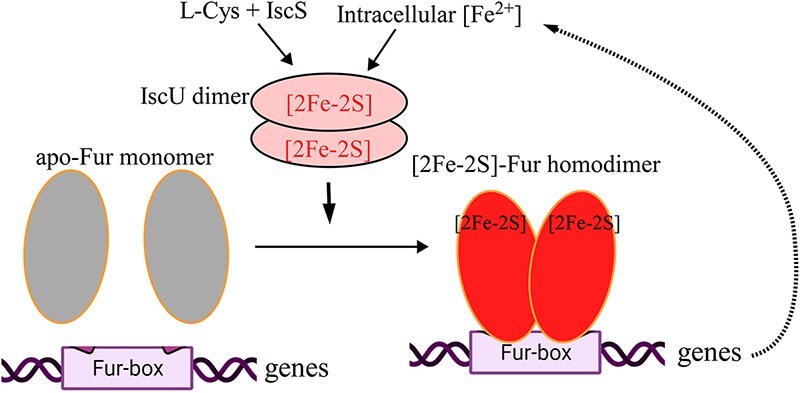

## Introduction

Iron-sulfur proteins have been discovered in diverse physiological processes ranging from energy metabolism to DNA replication and repair [1]. In *Escherichia coli*, there are at least 144 annotated iron-sulfur proteins, representing more than 3% of the *E. coli* proteome [2]. Because proteins may host iron-sulfur clusters via various amino acid residues [3], identifying new iron-sulfur proteins through bioinformatics has been challenging, and the exact number of iron-sulfur proteins in cells remains elusive. Nevertheless, increasing evidence suggests that iron-sulfur clusters in proteins are assembled by a group of highly conserved proteins [4, 5]. In *E. coli*, there are two major gene clusters: the house-keeping cluster *iscSUA-hscBA-fdx* for iron-sulfur cluster assembly in proteins under normal physiological conditions [4], and the stress-inducible cluster *sufABCDSE* [6] that are responsible for iron-sulfur cluster assembly in proteins under iron-starving [7] or oxidative stress [8] conditions. The iron-sulfur cluster assembly proteins recruit intracellular free iron content, extract sulfide from L-cysteine, assemble clusters, and deliver the clusters to target proteins [9]. An elevated intracellular iron content, however, is detrimental to cells, as excess ferrous iron promotes oxidative damage to cellular components via the Fenton reaction under aerobic conditions [10]. Thus, intracellular free iron content must be tightly regulated in such that there will be a sufficient amount of accessible iron for iron-sulfur cluster assembly without promoting oxidative damage in cells. In *E. coli* and many other bacteria, intracellular iron homeostasis is primarily controlled by a global transcription regulator Fur (ferric up regulator) [11–18]. Fur was initially described in *Salmonella typhimurium* in 1978 [12]. Since then, Fur had been characterized as an apo-repressor that became an active repressor upon binding its co-repressor Fe(II) [12–20]. Nevertheless, the proposed Fe(II)-bound Fur had only been reconstituted *in vitro* [13, 21, 22], and had never been identified *in vivo*. Serendipitously, we have found that the *E. coli* Fur binds a [2Fe-2S] cluster (instead of a mononuclear iron) to regulate intracellular iron homeostasis [23–27], and that the binding of the [2Fe-2S] cluster is highly conserved among Fur homologs from other bacteria [28]. This mini-review will present recent advances on the interplay between biogenesis of iron-sulfur clusters and regulation of intracellular iron homeostasis via the global transcription factor Fur in *E. coli*.

## Iron-sulfur cluster biogenesis and intracellular free iron content in *E. coli*

### Iron-sulfur cluster biogenesis and IscA

In *E. coli*, the house-keeping gene cluster *iscSUA-hscBA-fdx* encodes six core proteins: IscS (cysteine desulfurase) [29–31], IscU (a scaffold protein) [32–34], IscA (a proposed alternative scaffold protein) [35–38], HscB and HscA (two heat shock cognate proteins) [39–42], and ferredoxin (an electron carrier for iron-sulfur cluster assembly) [43, 44]. Cysteine desulfurase (IscS) catalyzes desulfurization of L-cysteine and delivers sulfide to scaffold protein IscU to form a nascent iron-sulfur cluster [32–34]. Scaffold protein IscU undergoes a concerted, sulfur-initiated [32] or an iron initiated [45] iron-sulfur cluster assembly process. HscB and HscA work together to promote the transfer of the assembled iron-sulfur cluster from IscU to target proteins during which ferredoxin provides electrons [9, 34, 39–42]. Among the six core cluster assembly proteins, much less is known on the function of IscA. Phylogenomic studies have shown that IscA is highly conserved among aerobic organisms and often has multiple paralogs in each organism [36]. For example, in *E. coli*, IscA homologs include ErpA [46–48], SufA [49], and NfuA [46]. IscA was initially characterized as an alternative scaffold for iron-sulfur cluster assembly, as purified IscA can assemble a [2Fe-2S] cluster *in vitro* [35, 38], and transfer the assembled clusters to target proteins [50, 51]. Interestingly, unlike the scaffold protein IscU, IscA has a strong and distinct binding activity for mononuclear iron [52–54] (see below).

The crystal structures of *E. coli* IscA [55, 56] did not provide information on the putative cluster binding site due to lack of electron density in the diffraction maps. On the other hand, the crystal structure of the *E. coli* IscA paralog, SufA, revealed a metal binding site that can potentially coordinate an iron-sulfur cluster or an iron atom [57]. Indeed, when co-expressed with operon *sufABCDSE, E. coli* SufA was purified as a [2Fe-2S] cluster-bound protein [49], demonstrating the iron-sulfur cluster binding activity of A-type proteins. Like *E. coli*, the cyanobacterium *Synechocystis* PCC 6803 has two IscA paralogs: IscA1 and IscA2. While IscA1 and IscA2 have 39% identity and 57% similarity, IscA1 binds a mononuclear iron and IscA2 binds a [2Fe-2S] cluster [58, 59], supporting the notion that IscA and its homologs are capable of binding an iron-sulfur cluster and a mononuclear iron atom.

In *E. coli*, deletion of IscA and its paralog SufA inhibits the [4Fe-4S] cluster assembly in endonuclease III and aconitase B but not the [2Fe-2S] cluster assembly in ferredoxin and SoxR [60], suggesting that IscA/SufA are essential for the [4Fe-4S] cluster assembly and dispensable for the [2Fe-2S] cluster assembly. Similarly, IscA and its paralog ErpA can partially replace each other in their role for the [4Fe-4S] cluster assembly in MoaA, a molybdenum cofactor synthase [61]. Furthermore, the IscA homologs ErpA and NfuA form a complex for iron-sulfur cluster delivery under oxidative stress conditions [62]. Like other iron-sulfur cluster assembly proteins, IscA is highly conserved from bacteria to humans [36], and human IscA1 can complement the function of IscA in *E. coli* cells [63]. In human and yeast mitochondria, IscA homologs (Isa1 and Isa2) are also required for the [4Fe-4S] cluster assembly (e.g. in aconitase) but not for the [2Fe-2S] cluster assembly (e.g. in ferrochelatase) [64, 65], and protein IBA57 recruits Isa2 for delivery of a [4Fe-4S] cluster to target proteins [66]. In plant mitochondria, IscA and NfU work together to deliver the [4Fe-4S] clusters to target proteins [67]. Thus, IscA and its paralogs have been proposed to act as carriers in a late-stage maturation of the [4Fe-4S] clusters in proteins [9, 68]. A caveat for this proposed carrier model is that IscA and its paralogs are completely dispensable for the [4Fe-4S] cluster assembly in proteins in *E. coli* cells under anaerobic conditions [60], suggesting that IscA and SufA are functional only under aerobic conditions [69]. Indeed, the genes encoding IscA and its homologs are mysteriously absent in all anaerobic organisms in which the [4Fe-4S] clusters are all assembled in proteins [36].

### A distinct iron binding activity of *E. coli* IscA

It has been well established that sulfide in iron-sulfur clusters is derived from L-cysteine via cysteine desulfurase (IscS) [29–31]. However, because cells have very low ‘free’ intracellular iron content [70], a dedicated iron chaperone is most likely required for iron-sulfur cluster biogenesis. A number of candidates have been proposed for the iron chaperone. CyaY, a bacterial homolog of frataxin, has been initially suggested as an iron donor for iron-sulfur cluster biogenesis [71–73]. While CyaY is capable of binding iron, the iron binding affinity is weak [74] and not sufficient to recruit iron from intracellular ‘free’ iron pool. Indeed, recent studies have suggested that instead of acting as an iron chaperone, CyaY/frataxin directly interacts with cysteine desulfurase IscS to regulate iron-sulfur cluster assembly in proteins [75, 76]. Another iron chaperone candidate is IscX [72, 77]. Like CyaY/frataxin, IscX also has a weak iron binding affinity, and the actual iron binding in IscX in cells has not been reported [77].

**Figure 1.**
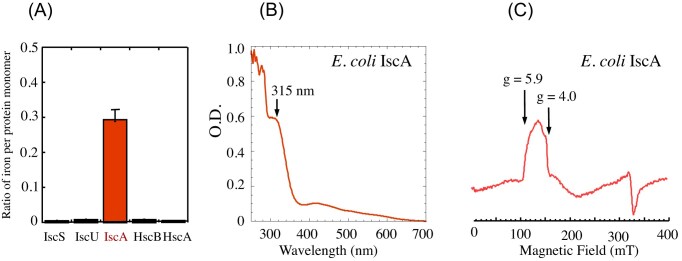
IscA is a strong iron binding protein in *E. coli* cells. **(A)**, iron content in iron-sulfur cluster assembly proteins purified from *E. coli* cells grown in LB medium under aerobic conditions. The ratio of iron to protein monomer is shown**. (B)**, UV-Vis absorption spectrum of IscA purified from *E. coli* cells grown in LB medium under aerobic conditions. The absorption peak at 315 nm reflects a mononuclear iron binding in IscA. **(C)**, EPR spectrum of as-purified iron-bound IscA. the *g* values at 5.9 and 4.0 indicate an S = 3/2 ferric iron center in IscA. (Adapted from [37] with permission from the royal society of chemistry).

When each iron-sulfur cluster assembly protein encoded by the *E. coli* iron-sulfur cluster assembly cluster *iscSUA-hscBA-fdx* [4] was expressed in *E. coli* cells grown in LB medium under aerobic conditions, only purified IscA contained a significant amount of mononuclear iron [52] (Fig. 1A). The UV-Vis absorption measurements of purified IscA revealed a 315 nm peak (Fig. 1B), indicative of the mononuclear iron binding, which is very similar to that of the cyanobacterium *Synechocystis* PCC 6803 IscA1 [58, 59]. The electron paramagnetic resonance (EPR) measurements showed that IscA bound a high-spin 3/2 Fe(III) center (Fig. 1C) which was readily released by L-cysteine for iron-sulfur cluster assembly in proteins [37, 69].

When apo-form IscA was incubated with a stoichiometric amount of Fe(II) in the presence of thioredoxin/thioredoxin reductase [78], IscA bound Fe(II) with a dissociation constant of 3.0 × 10^19^ M^−1^ [52]. Apo-form IscA was also capable of retrieving iron from ferritin A and delivering the iron for iron-sulfur cluster assembly in proteins *in vitro* [53]. Under aerobic conditions, Fe(II) was quickly oxidized and became inaccessible for iron-sulfur cluster assembly in proteins. The IscA-bound Fe(II), however, was stable and fully accessible for the iron-sulfur cluster assembly in proteins under aerobic conditions [69]. Similarly, Johnson’s group reported that the ^Nif^IscA homolog from *Azotobacter vinelandii* could not only bind an iron-sulfur cluster [79] but also a mononuclear iron and provide iron for the iron-sulfur cluster assembly on the N-terminal domain of NifU [54]. Collectively, these results suggested that IscA may act as an iron chaperone that recruits intracellular free iron and delivers the iron for iron-sulfur cluster assembly in proteins under aerobic conditions.

If IscA is an iron donor for iron-sulfur cluster biogenesis, deletion of IscA and its paralog SufA would affect both the [2Fe-2S] cluster and [4Fe-4S] cluster assembly in proteins in cells. However, as described above, deletion of IscA and SufA only inhibits the [4Fe-4S] cluster assembly in proteins in *E. coli* [60], yeast [65], and human [64] cells. One possible explanation is that the [2Fe-2S] clusters and [4Fe-4S] clusters in protein have distinct assembly mechanisms. A recent study indicated that apo-form ferredoxin (a [2Fe-2S] cluster-binding protein) binds a mononuclear iron *in vitro* and in *E. coli* cells grown at low temperature [80]. On the other hand, aconitase B (a [4Fe-4S] cluster-binding protein) does not have any iron binding activity under the same experimental conditions, suggesting that the [2Fe-2S] clusters and the [4Fe-4S] cluster are assembled in distinct mechanisms. In this context, we propose that IscA (and its paralogs) delivers iron for the [4Fe-4S] cluster assembly in proteins under aerobic conditions [60, 61, 64, 65].

### Deletion of IscA/SufA results in accumulation of the persulfide-bound IscS in *E. coli* cells under aerobic growth conditions

Iron-sulfur clusters are assembled in proteins by a coordinated delivery of iron [45] and sulfide [32] to the scaffold protein IscU [32–34]. A physical interaction between IscU and cysteine desulfurase IscS which delivers sulfide for iron-sulfur cluster assembly in IscU has been demonstrated [81, 82]. If IscA is an iron chaperone, IscA may also have physical interactions with IscU and/or IscS. However, so far, there has been no evidence suggesting that IscA has any physical interactions with IscU or IscS.

**Figure 2.**
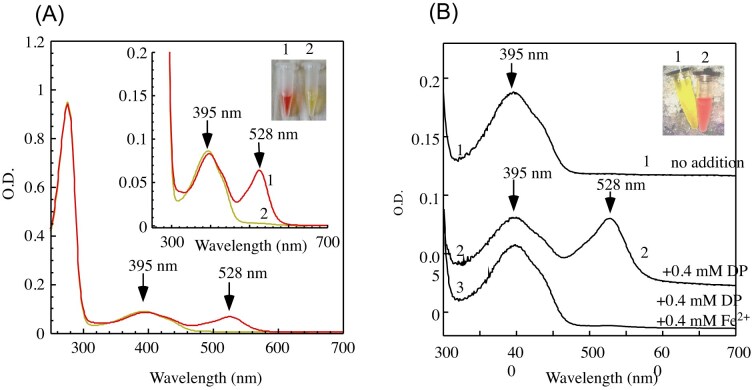
Accumulation of the persulfide-bound IscS in *E. coli* mutant cells with deletion of IscA/SufA or depletion of intracellular free iron using 2,2’-dipyridyl. A), IscS was expressed in *iscA/sufA* mutant (spectrum 1) or wild-type *E. coli* (spectrum 2) cells. B), IscS was expressed in wild-type *E. coli* cells (spectrum 1), wild-type cells supplemented with 2,2’-dipyridyl (0.4 mM) (spectrum 2) or 2,2’-dipyridly (0.4 mM) and Fe(NH_4_)_2_(SO_4_)_2_ (0.4 mM) (spectrum 3). (Adapted from [83]. © the American Society for Biochemistry and Molecular Biology).

To explore the physiological interactions between IscA and IscS/IscU, we expressed IscU in the *E. coli* mutant in which IscA and its paralog SufA were deleted. UV-visible absorption spectra of IscU purified from wild-type and the *iscA/sufA* mutant cells were essentially identical. On the other hand, when IscS was expressed in the *iscA/sufA* mutant cells, purified IscS had a red color, while IscS purified from wild-type *E. coli* cells had a typical yellow (pyridoxal 5'-phosphate) color (Fig. 2A) [83]. Additional studies revealed that the red-color IscS had persulfate and was only partially active to catalyze L-cysteine desulfurization [83]. If IscA provides iron for the iron-sulfur cluster biogenesis under aerobic conditions as proposed [60], deletion of IscA and its paralog SufA will block the iron delivery, resulting in accumulation of the persulfide-bound IscS (Fig. 2A). To test this idea, we expressed IscS in the wild-type *E. coli* cells in which intracellular free iron was depleted using a membrane permeable iron chelator 2,2’-dipyridyl. The results showed that the addition of 2,2’-dipyridyl produced the same persulfide-bound IscS with an absorption peak at 528 nm (Fig. 2B).

Thus, deletion of IscA/SufA or depletion of intracellular free iron content leads to the same accumulation of the persulfide-bound IscS in *E. coli* cells [83], further suggesting that IscA acts as an iron chaperone for the [4Fe-4S] cluster biogenesis in *E. coli* cells.

**Figure 3.**
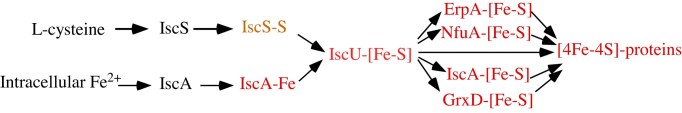
A proposed model for the interplay between IscA and IscS in *E. coli* cells under normal growth conditions. IscS catalyzes desulfurization of L-cysteine and IscA recruits intracellular free iron for iron-sulfur cluster biogenesis. Deletion of IscA or depletion of intracellular free iron content results in the accumulation of the persulfide-bound red IscS. The iron-sulfur cluster assembly carriers including IscA, ErpA, NfuA, and GrxD deliver the assembled [4Fe-4S] clusters to target proteins.

Figure 3 summarizes the [4Fe-4S] cluster assembly in *E. coli* cells under aerobic growth conditions where sulfur is derived from L-cysteine by IscS and intracellular free iron is recruited by IscA. Sulfur and iron are coordinately delivered to the scaffold protein IscU. The iron-sulfur cluster assembled in IscU is then transferred to target proteins via carriers including Nfu [62, 84, 85], IscA [61], ErpA [62], and monothiol glutaredoxin (GrxD) [46].

### Deletion of IscA and SufA elevates intracellular ‘free’ iron content in *E. coli*

If IscA is an iron chaperone for the [4Fe-4S] cluster assembly under aerobic conditions, deletion of IscA and its homologs will lead to elevation of intracellular free iron content. Indeed, in *Saccharomyces cerevisiae* cells, deletion of Isa1 and Isa2 resulted in accumulation of iron in mitochondria and exhibited a dependency on lysine and glutamate for cell growth [86]. Similarly, in *E. coli* cells, deletion of IscA and SufA significantly elevated intracellular free iron content [23].

## The ferric uptake regulator (Fur) binds a [2Fe-2S] cluster in response to elevation of intracellular free iron content in *E. coli*

### The ferric uptake regulator (Fur) regulates intracellular iron homeostasis

The ferric uptake regulator (Fur) is a global transcription factor that controls over 150 genes responsible for intracellular iron transportation and storage, energy metabolism, oxidative stress, and bacteria virulence [12–20]. The Fur-regulated gene’s promoters have an operator site with 19-bp inverted repeats (5’-GATAATGATAATCATTATC-3’) known as the Fur-box [87, 88]. Binding of an active Fur to the Fur-box occludes the RNA polymerase binding site and represses transcription initiation of target genes including those encoding iron transportation proteins FepA and Fes [11, 15, 16]. In *Neisseria gonorrhoeae*, active Fur may also act as a transcriptional activator by binding to the upstream of the promoter and recruiting RNA polymerase [89, 90]. Likewise, apo-form Fur may repress the expression of genes in *Helicobacter pylori* [91, 92] or activate the expression of genes in *vibrio vulnificus* [93]. Furthermore, a number of the Fur-regulated genes are indirectly activated via a small regulatory RNA RyhB [94, 95]. Expression of RyhB is repressed by active Fur, and production of RyhB in turn promotes the degradation of mRNAs of over 56 genes in *E. coli* cells [96, 97]. In addition, some genes (e.g. the ferritin A gene) are activated by active Fur via displacing the repressor H-NS in the operator site [98]. Thus, Fur regulates the expression of a broad range of genes via various regulatory mechanisms (see the reviews [11, 16, 99, 100]).

### The current model for the regulation of intracellular iron homeostasis by Fur

**Figure 4.**
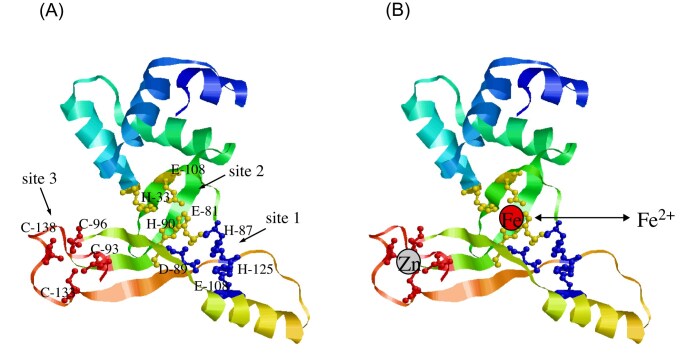
A current model for activation of *E. coli* Fur. **(A)**, the proposed metal binding sites of Fur. Site 1 is coordinated by His-87, Asp-89, Glu-108, and His-125 (indicated in blue color); site 2 is coordinated by His-33, Glu-81, His-88, and His-90 (indicated in yellow color); site 3 is coordinated by Cys-93, Cys-96 (indicated in red color). The full-length *E. coli* Fur structure model was constructed using Alphafold2. **(B)**, the current model for Fur activation. An active Fur has a structural Zn(II) binding at site 3 and a regulatory Fe(II) binding at site 2. Site 1 is considered an accessory site.

Since its discovery in 1980s [12], Fur had been characterized as an apo-repressor that binds its co-repressor Fe(II) to regulate the expression of its target genes [12–20]. The assumption that Fur binds Fe(II) has been widely accepted [19, 101] and used to model the metalation of proteins [102, 103] in bacteria. Indeed, the *in vitro* studies showed that apo-form Fur can bind divalent transition cations Fe(II), Mn(II), Zn(II), Cu(II), and Co(II), and become an active repressor to bind the Fur-box [13, 21, 22], except that the Zn(II)-bound Fur appears to be inactive to repress the expression of target genes in *E. coli* cells [13, 104]. Crystallographic studies of *E. coli* Fur [105] and its homologs from other bacteria [106–111] revealed that each Fur monomer has three metal binding sites: site 1 (coordinated by His-87, Asp-89, Glu-108, and His-125) is located within the dimerization domain; site 2 (coordinated by His-33, Glu-81, His-88, and His-90) connects the DNA binding domain and the dimerization domain; and site 3 (coordinated by Cys-93, Cys-96) is at the C-terminal end of the dimerization domain [105, 108]. In some cases (e.g. *C. jejuni* Fur [110]), site 1 in Fur is termed site 3, and site 3 is site 1. For simplicity, we will follow the metal binding site numbers in *E. coli* Fur (Fig. 4A). Since the available crystal structure of *E. coli* Fur only has the N-terminal region of the amino acid residues 1-82 [105], the full-length *E. coli* Fur crystal structure model is constructed using the Alphafold2 [112] and is shown in Fig. 4A.

In the *Pseudomonas aeruginosa* Fur crystal structure, site 1 was proposed as a regulatory site while site 2 a structural one [106]. Nevertheless, in the crystal structure of the *Francise tularensis* Fur, it binds Fe(II) at site 2 and Zn(II) at site 3 [111] (Fig. 4B). Furthermore, the Mössbauer and EXAFS spectroscopic studies indicated that *E. coli* Fur binds Fe(II) at site 2 and Zn(II) at site 3 [113], suggesting that site 3 is a Zn(II)-bound structure site and site 2 is a regulatory site. However, both the *F. tularensis* Fur [111] and the *E. coli* Fur [113] were reconstituted with excess Fe(II) *in vitro* under anaerobic conditions. Because the dissociation constants of the *E. coli* Fur for Mn(II), Fe(II), Co(II), and Cu(II) are in the range of 10 to 85 μM *in vitro* [21, 22], and the intracellular free iron concentration in *E. coli* cells is less than 1 μM [70], it is almost impossible for Fur to bind any Fe(II) in *E. coli* cells. Indeed, so far, no evidence is available to support the hypothesis that Fur binds Fe(II) in any bacteria.

One argument for the mononuclear iron binding in Fur protein came from the observation that when Fur in *E. coli* cells was exposed to nitric oxide (NO) gas, Fur formed a dinitrosyl iron complex [114, 115]. However, the formation of dinitrosyl iron complex in proteins does not require a mononuclear iron in protein. An iron-sulfur cluster can also be readily modified by NO gas forming a protein-bound dinitrosyl iron complex *in vitro* and *in vivo* [116, 117].

When *E. coli* Fur was expressed in *E. coli* cells grown in LB medium, purified Fur contained two Zn(II) atoms per monomer, one of them was loosely bound, and the other tightly bound [104, 105, 118]. Chemical modification and mass spectrometry analysis showed that Fur bound the tight Zn(II) at site 3 via two cysteine residues (Cys-93 and Cys-96) and two other unknown ligands (one of them is most likely a histidine), and became a homodimer [118–120]. The tight Zn(II) binding at site 3 was also found in the *F. tularensis* Fur [111] and *H. pylori* Fur [120] via four cysteine residues. While the Fur with the tightly bound Zn(II) was active to bind the Fur-box, the binding of weakly bound Zn(II) or Fe(II) at site 2 apparently enhanced the Fur-box binding activity of Fur [104], suggesting that the repressor activity of Fur could be regulated by the metal binding at site 2 (Fig. 4B).

### *The E. coli* Fur binds a [2Fe-2S] cluster in the *E. coli* mutant cells with deletion of IscA and SufA

**Figure 5.**
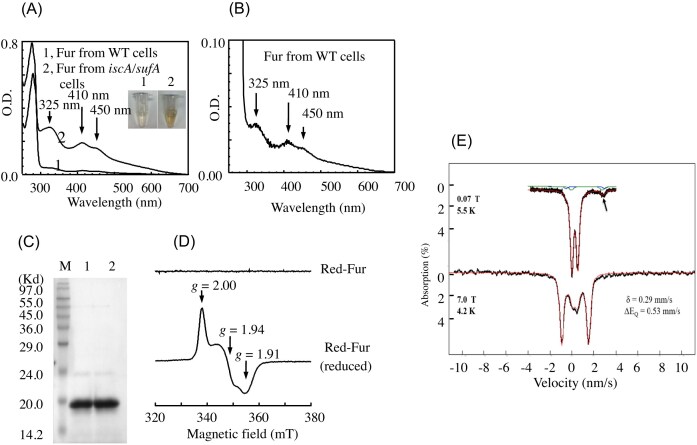
The *E. coli* Fur binds a [2Fe-2S] cluster. *E. coli* Fur was purified from the *E. coli* mutant cells with deletion of IscA/SufA. **(A)**, Fur purified from wild-type (spectrum 1) and *iscA/sufA* mutant cells (spectrum 2). insert is a photograph of purified fur proteins from wild type and the *iscA/sufA* mutant cells. **(B)**, expanded spectrum of Fur purified from wild-type cells. **(C)**, SDS-PAGE gel of Fur proteins purified from wild-type and the *iscA/sufA* mutant cells. **(D)**, EPR spectra of Fur purified from the *iscA/sufA* mutant cells before and after reduction with sodium dithionite. **(E)**, Mössbauer spectra of Fur purified from the *iscA/sufA* mutant cells. Top spectrum, at magnetic field of 0.07 T at 5.5 K. Botton spectrum, at magnetic field of 7.0 T at 4.2 K. (Adapt from [23]. © the american society for biochemistry and molecular biology).

Since the *E. coli* mutant with deletion of IscA/SufA has an elevated intracellular free iron content [23, 60], we reasoned that the *iscA/sufA* mutant cells may provide an opportunity to explore the Fe(II)-bound Fur *in vivo*. When Fur was expressed in the *E. coli iscA/sufA* mutant cells grown in LB medium under aerobic conditions, purified Fur exhibited a bright red color. The UV-Visible (Fig. 5A), Electron Paramagnetic Resonance (EPR) (Fig. 5D), and Mössbauer (Fig. 5E) studies showed that red Fur bound a unique [2Fe-2S] cluster, but not a mononuclear iron center [23]. Quantification of iron and sulfide content showed that about ∼36% of red Fur protein contained a [2Fe-2S] cluster.

Fur was also expressed in wild-type *E. coli* cells grown in LB medium under aerobic conditions. In this case, only about ∼8% of Fur contained a [2Fe-2S] cluster (Fig. 5B), indicating that the binding of the [2Fe-2S] cluster in Fur is also present in the wild-type *E. coli* cells, and that deletion of IscA and SufA elevates intracellular free iron content and promotes the [2Fe-2S] cluster binding in Fur in *E. coli* cells.

### Fur progressively binds a [2Fe-2S] cluster in wild-type *E. coli* grown in M9 medium supplemented with increasing concentrations of iron

**Figure 6.**
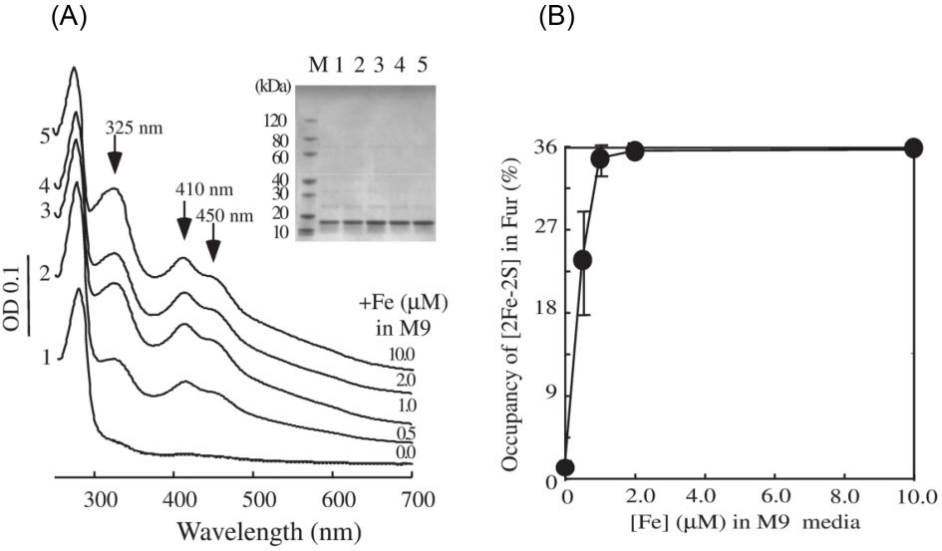
The *E. coli* Fur progressively binds a [2Fe-2S] cluster in response to elevation of intracellular free iron content. **(A)** UV-Vis absorption spectra of Fur purified from wild-type *E. coli* cells grown in M9 medium supplemented with 0.0 (1), 0.5 (2), 1.0 (3), 2.0 (4), or 10.0 (5) μM Fe(NH_4_)_2_(SO_4_)_2_ under aerobic growth conditions. Purified Fur (50 μM) were in buffer containing NaCl (500 mM) and Tris (20 mM, pH 8.0). Insert is a photograph of the SDS-PAGE gel of purified Fur proteins. **(B)** the *E. coli* Fur binds the [2Fe-2S] cluster in wild-type *E. coli* cells in response to increasing concentrations of iron in M9 medium. The [2Fe-2S] cluster occupancies of Fur proteins purified from wild-type *E. coli* cells grown in M9 medium were plotted as a function of the iron concentrations in the medium. Data represents the averages ± standard deviations from three independent experiments. (Adapted from [24], under the Creative Commons CCBY 4.0 License).

Unlike LB medium which contains significant amounts of Zn(II) and other divalent transition cations, M9 medium has the defined ingredients and very limited amounts of divalent transition cations [121]. When *E. coli* Fur was expressed in the wild-type *E. coli* cells grown in M9 medium supplemented with iron, Fur progressively bound a [2Fe-2S] cluster as the iron concentration in M9 medium increased from 0 to 10 μM (Fig. 6A). About 20% of Fur bound a [2Fe-2S] cluster when *E. coli* cells were grown in M9 medium supplemented with 0.5 μM iron. The occupancy of the [2Fe-2S] cluster in Fur increased to 36% when the *E. coli* cells were grown in M9 medium supplemented with 1.0 μM iron. Further increase of iron concentration in M9 medium did not increase the [2Fe-2S] cluster occupancy in Fur in *E. coli* cells (Fig. 6B) [24], likely due to the intracellular iron homeostasis regulation by Fur. The whole-cell Mössbauer spectroscopy studies revealed that Fur bound an oxidized [2Fe-2S] cluster but no mononuclear iron in *E. coli* cells [26]. The ICP-MS analyses of the purified Fur protein further confirmed that Fur only bound a [2Fe-2S] cluster without additional Zn(II) or Fe(II) [27].

To test whether the binding of a [2Fe-2S] cluster is conserved among Fur proteins, we expressed the Fur homologs from *Haemophilus influenzae, Vibrio cholera*, and *H. pylori* in the *E. coli* cells with an elevated intracellular free iron content. Like the *E. coli* Fur, all these Fur homologs are capable of binding a [2Fe-2S] cluster in response to elevation of intracellular free iron content [28]. Interestingly, the *H. influenzae* Fur has the strongest binding affinity for the [2Fe-2S] cluster among these Fur homologs, while other Fur proteins have similar binding activities for the cluster [28], suggesting that the *H. influenzae* Fur may have its distinct regulation of intracellular iron homeostasis. Recently, the Fur homolog from *Acidithiobacillus ferrooxidans* has also been shown to bind an iron-sulfur cluster [122], supporting the idea that the iron-sulfur cluster binding is highly conserved among Fur proteins.

### Fur becomes an active repressor upon the binding of a [2Fe-2S] cluster

**Figure 7.**
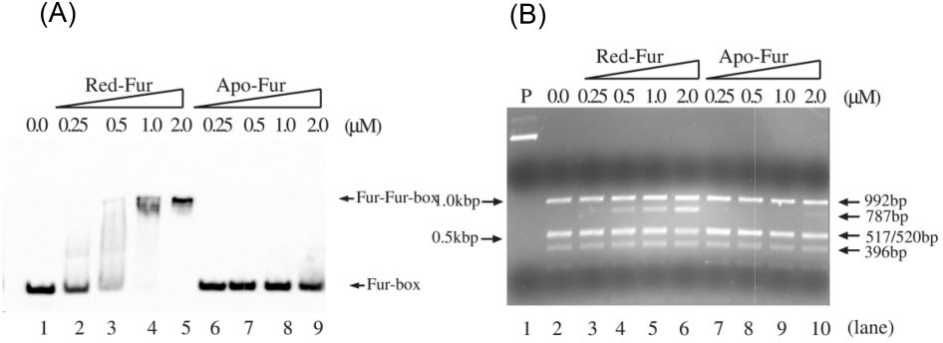
The Fur-box binding activity of the [2Fe-2S] cluster-bound Fur and apo-Fur. **A)**, band shift assays of red-Fur and apo-Fur. Biotin-labeled Fur-box DNA (0.7 nM) was incubated with the indicated concentrations of red-Fur or apo-Fur. lane 1, no Fur protein. lanes 2 to 5, biotin-labeled Fur-box DNA (0.7 nM) was incubated with 0.25, 0.5, 1.0, and 2.0 μM red-Fur, respectively. lanes 6 to 9, biotin-labeled Fur-box DNA (0.7 nM) was incubated with 0.25, 0.5, 1.0, and 2.0 μM apo-Fur, respectively. **B,)** the restriction site protection assays of red-Fur and apo-Fur. pUC18-*iuc* (3.2 nM) was pre-incubated with increasing concentrations of red-Fur and apo-Fur, followed by digestion with *hin*fI (1 unit) at 37 °C for 10 min. the digested DNA products were separated by 1.5% agarose gel electrophoresis. lane 1, pUC18-*iuc* only. lane 2, no Fur protein was added. lanes 3 to 6, pUC18-*iuc* (3.2 nM) was pre-incubated with 0.25, 0.5, 1.0, and 2.0 μM red-Fur, respectively. lanes 7 to 10, pUC18-*iuc* was pre-incubated with 0.25, 0.5, 1.0, and 2.0 μM apo-Fur, respectively. (Adapted from [24], under the creative commons CCBY 4.0 license).

As a repressor, Fur represses the expression of its target genes by binding to specific DNA sequences known as Fur-box in the promoter region [87, 88]. To test whether Fur is active to bind the Fur-box upon the binding of a [2Fe-2S] cluster, we prepared a consensus Fur-box (5’-TATAATGATACGCATTATC-3’) [87] labeled with biotin and incubated with increasing concentrations of apo-Fur or the [2Fe-2S] cluster-bound Fur, followed by the electrophoretic mobility shift assays. Figure 7A shows that while apo-Fur (Fur without [2Fe-2S] cluster or Zn(II) bound) had no binding activity of the Fur-box, purified red Fur shifted the Fur-box DNA. Because only about 36% of red Fur protein contained a [2Fe-2S] cluster (Fig. 6), the ‘pure’ [2Fe-2S] cluster-bound Fur likely has the comparable binding activity for the Fur-box as the previously reported metal-bound Fur [104, 111, 120].

To further explore the Fur-box binding activity of apo-Fur and red Fur, we used the restriction site protection assay as described in [24]. The promoter region of the operon *iucABCD* which encodes the enzymes for biosynthesis of siderophore aerobactin has a consensus Fur-box sequence (5’-GAGAATCATTAGCATTCGC-3’) that also contains the restriction *hin*fI site (5’-GANTC-3’) [13]. The promoter region of the operon *iucABCD* was cloned into plasmid pUC19 to create pUC19-*iuc* [24]. This approach has been used to determine the Fur-box binding of *E. coli* Fur after nitric oxide exposure [114], of the Co(II)-bound *H. pylori* Fur [120], and of the Mn(II)-bound *Aliivibrio salmonicida* Fur [123]. Figure 7B shows that red Fur protected the Fur-box in pUC19-*iuc* from the *Hin*fI digestion (lane 5). In contrast, apo-Fur (up to 2.0 μM) had no protection for the Fur-box in pUC19-*iuc* (lane 7). Taken together, the results demonstrated that unlike apo-Fur, the [2Fe-2S] cluster-bound Fur is active to bind the Fur-box *in vitro*.

### Fur binds a [2Fe-2S] cluster to form a homodimer via the conserved cysteine residues at the C-terminus.

Structure studies have shown that Fur has three putative metal binding sites (Fig. 4A). To investigate where the [2Fe-2S] cluster binds in the *E. coli* Fur, we expressed the C-terminal domain of the *E. coli* Fur (residues 83-148) in the *E. coli* cells with an elevated intracellular free iron content, and found that the C-terminal domain was sufficient for binding a [2Fe-2S] cluster [124]. We then constructed Fur mutants in which Cys-93, Cys-96, or Cys-133 was replaced with Ala, and found that Fur proteins with mutations of Cys-93, Cys-96, or Cys-133 to Ala (site 3) did not bind any [2Fe-2S] cluster [24]. On the other hand, mutations at site 1 (Glu-108 to Ala) or site 2 (His-90 to Ala) had no effects on the [2Fe-2S] cluster binding in the *E. coli* Fur. Thus, Fur binds a [2Fe-2S] cluster at site 3 via the cysteine residues [23].

**Figure 8.**
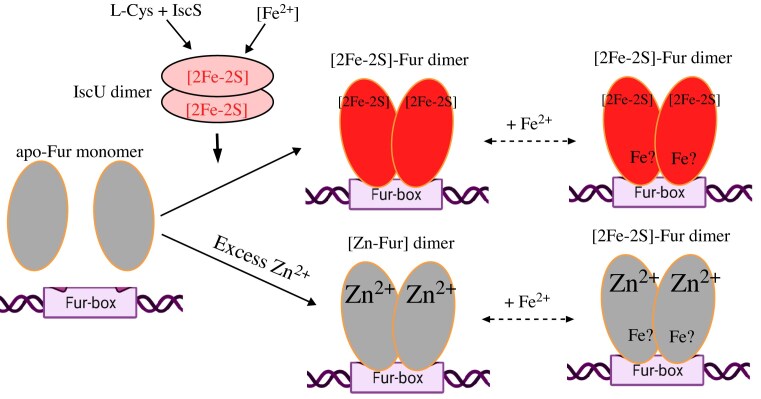
A new model for the activation of the *E. coli* Fur. Apo-form Fur is inactive to bind the Fur-box. Upon the binding a [2Fe-2S] cluster at site 3, Fur becomes a homodimer to bind the Fur-box. Fur can also bind Zn(II) at the same binding site (site 3) when the intracellular Zn(II) content is elevated and become a homodimer to bind the Fur-box. The binding of Zn(II) effectively blocks Fur to sense intercellular free iron content. Without concrete evidence, both Fur might also bind Fe(II) at site 2 to enhance the Fur-box binding activity. (Adapted from [27] under the Creative Commons CCBY 4.0 License).

Since *E. coli* Fur [104, 105, 118] and Fur from other bacteria [111, 120] also bind a tight Zn(II) at site 3, Fur likely binds the [2Fe-2S] cluster [23–26, 28] and Zn(II) [118–120] at the same site (site 3). Gel filtration analyses showed that *E. coli* Fur became a homodimer upon binding of a [2Fe-2S] cluster or Zn(II) at site 3 [27]. While mutations at site 1 and site 2 had no effects on the [2Fe-2S] cluster binding and Zn(II) binding in Fur, mutation at site 3 completely eliminated both the [2Fe-2S] cluster binding and the Zn(II) binding in Fur [27]. Indeed, Zn(II) could effectively compete for the [2Fe-2S] cluster binding in Fur in *E. coli* cells [27]. It appears that in LB medium in which Zn(II) is abundant, native levels of Fur may be targeted under high zinc stress by Zn(II) binding to site 3 (displacement of the [2Fe-2S] cluster) in Fur in the wild-type *E. coli* cells [104, 105, 118]. In M9 medium in which Zn(II) and other divalent transition cations are limited, Fur binds a [2Fe-2S] cluster in response to elevation of intracellular free iron content to regulate intracellular iron homeostasis [23–26, 28]. When there are excess divalent transition cations in M9 medium, these transition cations will compete for the [2Fe-2S] cluster binding site in Fur. Although the Zn(II)-bound Fur and the [2Fe-2S] cluster-bound Fur have similar binding activities for the Fur-box *in vitro*, it is likely that they will have distinct binding properties for the Fur-box [13, 104]. Regardless, we propose that the binding of Zn(II) at site 3 in Fur will effectively block Fur to sense intracellular free iron content, therefore disrupting iron homeostasis in bacteria (Fig. 8).

The intactness of site 1 or site 2 is also likely crucial for the structure and function of Fur [108, 109]. While the ICP-MS and Mössbauer measurements of the purified [2Fe-2S] cluster-bound *E. coli* Fur and the whole cell Mössbauer spectroscopy showed that *E. coli* Fur only binds a [2Fe-2S] cluster with no additional metal binding [26], we could not exclude the possibility that the [2Fe-2S] cluster-bound Fur binds Fe(II) at site 2 as previously proposed [111] (Fig. 8). However, the weak Fe(II) binding activity of Fur [21, 22] and extremely low intracellular free iron concentrations in *E. coli* cells [70] would prevent any significant Fe(II) binding in Fur. Furthermore, the binding of a [2Fe-2S] cluster at site 3 is necessary and sufficient for the Fur-box binding activity of Fur in response to elevation of intracellular free iron content. Nevertheless, additional experiments (e.g. the *in vitro* transcription assay to demonstrate that the [2Fe-2S] cluster-bound Fur actually represses the transcription initiation) are needed to prove that the [2Fe-2S] cluster-bound Fur is a bona fide transcription repressor in cells.

It should be pointed out that some Fur homologs such as the *Pseudomonas aeruginosa* Fur do not have the corresponding cysteine residues that host the [2Fe-2S] cluster [125]. Whether these Fur homologs without the cysteine residues are capable of binding a [2Fe-2S] cluster or use other unknown ways to sense intracellular free iron content remains to be further investigated.

### *The E. coli* Fur is a new member of the transcription factor family that binds an iron-sulfur cluster

Use of an iron-sulfur cluster to sense intracellular ‘free’ iron content is not unprecedented. In mammalian cells, the iron regulatory protein 1 (IRP-1) regulates the intracellular ‘free’ iron content via reversible binding of a [4Fe-4S] cluster in response to an elevated intracellular ‘free’ iron content [126]. In yeast cells, the iron sensors Yap5 [127, 128] and Aft2 [129] in *Saccharomyces cerevisiae* and Fep1 [130] in *Pichia pastoris* also bind a [2Fe-2S] cluster in response to an elevated intracellular ‘free’ iron content. Thus, the *E. coli* Fur is a new example of transcription factors that regulate intracellular iron homeostasis via binding a [2Fe-2S] cluster [23].

In bacteria, a growing number of transcription factors use an iron-sulfur cluster as a sensor [131, 132]. The *E. coli* anaerobic regulator FNR binds an oxygen sensitive [4Fe-4S] cluster to regulate the expression of genes involved in anaerobic metabolism [133]; the *E. coli* IscR binds a [2Fe-2S] cluster in response to an elevated level of iron-sulfur cluster assembly activity and represses the expression of the iron-sulfur cluster assembly genes [134]; the *E. coli* SoxR uses a [2Fe-2S] cluster to sense the intracellular redox potential [135, 136]; the kinase NreB of *Staphylococcus carnosus* uses an oxygen labile [4Fe-4S] cluster to sense oxygen [137]; and the repressor NsrR of *Streptomyces coelicolor* uses a [4Fe-4S] cluster to sense nitric oxide (NO) [138]. The *E. coli* Fur represents a new member of the iron-sulfur cluster-containing transcription factor family and links intracellular iron homeostasis with iron-sulfur cluster biogenesis in bacteria.

## Iron-sulfur cluster biogenesis and regulation of Fur in *E. coli*

### The [2Fe-2S] cluster in Fur is assembled by the iron-sulfur cluster assembly machinery

The finding that the *E. coli* Fur binds a [2Fe-2S] cluster in response to elevation of intracellular ‘free’ iron content in *E. coli* cells suggests that the [2Fe-2S] cluster in Fur may be assembled by the iron-sulfur cluster assembly machinery [4]. However, deletion of IscA and SufA promotes the [2Fe-2S] cluster assembly in Fur in *E. coli* cells grown in LB medium [23], suggesting that IscA and SufA are not directly involved in the [2Fe-2S] cluster assembly in Fur. Because IscS provides sulfur not only for the iron-sulfur cluster biogenesis [31] but also for other biosynthesis pathways [139–142], deletion of IscS will have multiple physiological consequences. We therefore constructed an *E. coli* mutant in which IscU was deleted. When Fur was expressed in the *E. coli iscU* mutant cells grown in M9 medium supplemented with iron, very little or no [2Fe-2S] cluster was assembled in Fur, suggesting that IscU was required for the [2Fe-2S] cluster assembly in Fur in *E. coli* cells [25]. The results also suggested that the [2Fe-2S] cluster in Fur was enzymatically assembled in *E. coli* cells. The enzymatic assembly of the [2Fe-2S] cluster in Fur (rather than thermodynamic binding of Fe(II) to Fur in cells) may meet the requirements of the repressor to rapidly sense the fluctuation of intracellular free iron content and regulate iron homeostasis in bacteria.

### The expression of gene *fur* is regulated by the iron-sulfur cluster biogenesis

The expression of gene *fur* in *E. coli* is regulated by the oxidative stress regulatory systems, OxyR and SoxRS [143], and by the Fur itself [144]. SoxR is a [2Fe-2S] cluster binding protein [135, 136], and oxidation of the [2Fe-2S] cluster in SoxR switches on its transcription activity to stimulate the expression of transcription factor SoxS which activates the expression of the gene *fur* and its immediate upstream gene *fldA* [143]. In addition, translation of the *fur* mRNA is coupled to that of an upstream open reading frame (uof) [144], and the stability of the *uof-fur* mRNA is regulated by a small regulatory RyhB [95]. Expression of RyhB is negatively regulated by Fur [95], and RyhB interacts with the *uof* and blocks translation of the down-stream mRNA *fur* [144]. When there is an iron deficiency, Fur loses its [2Fe-2S] cluster and de-represses the expression of RyhB. Expression of RyhB inhibits the translation of Fur mRNA and prevents excess production of Fur in *E. coli* cells [95]. Since both SoxR and Fur are a [2Fe-2S] cluster binding protein [23], the iron-sulfur cluster biogenesis will directly affect the expression of gene *fur*.

Recent studies also suggested that TusA, a protein involved in both iron-sulfur cluster biogenesis and molybdenum cofactor biosynthesis [145], may have a regulatory role for the Fur activation [146]. TusA has a specific interaction with cysteine desulfurase IscS [76]. Deletion of TusA results in less active Fur in *E. coli* [146], implying that TusA may contribute to regulation of Fur via iron-sulfur cluster biogenesis.

### Fur regulates the expression of the iron-sulfur cluster assembly machineries

As a global transcription factor, Fur regulates over 150 genes in *E. coli* genome [11]. Among the Fur-regulated genes are the ones encoding iron-sulfur cluster assembly proteins: the house-keeping *iscSUA-hscBA-fdx* cluster [4] and the stress-inducible *sufABCDSE* cluster [6, 7].

**Figure 9.**
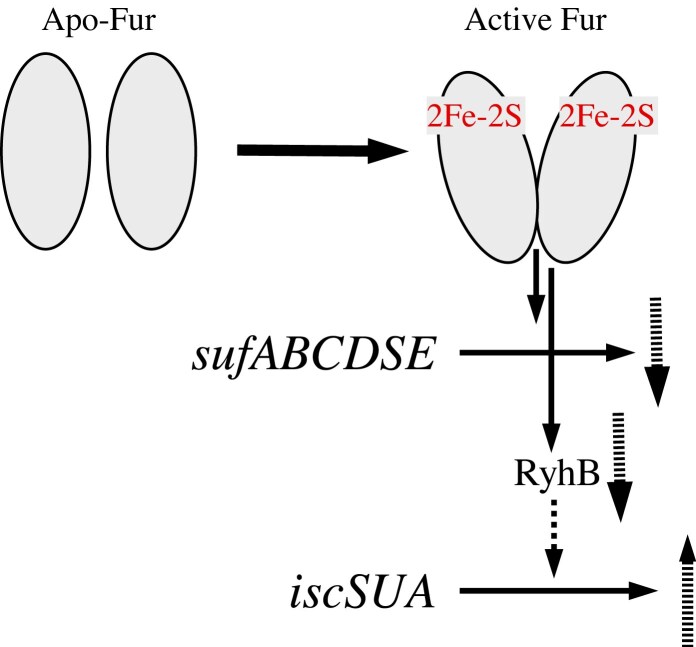
Regulation of iron-sulfur cluster biogenesis by Fur in *E. coli*. Fur becomes active to repress the expression of its target genes *sufABCDFE* and the regulatory small RNA RyhB. Decreased expression of RyhB stabilizes *iscSUA* mRNA and increases the expression of IscS, IscU, and IscA for the house-keeping iron-sulfur cluster biogenesis.

The expression of the *iscSUA-hscBA-fdx* cluster in *E. coli* is indirectly regulated by Fur via the regulatory RNA RyhB [144] which binds to the *iscSUA* mRNA and promotes the degradation of the *iscSUA* mRNA [147]. When intracellular free iron content is elevated, Fur represses the expression of RyhB [24], thus promoting iron-sulfur cluster assembly activity of the *iscSUA-hscBA-fdx* cluster. When intracellular free iron content is scarce, Fur loses the [2Fe-2S] cluster, and RyhB is highly expressed to degrade the *iscSUA* mRNA and prevents the production of unnecessary iron-sulfur cluster assembly activity [147] (Fig. 9).

The expression of the inducible *sufABCDSE* cluster is directly regulated by Fur, as the promoter of the cluster contains a Fur-box site [7]. The active Fur binds the promoter region of the *sufABCDSE* cluster and blocks the transcription of *sufABCDSE* (Fig. 9). Thus, when intracellular iron content is elevated, Fur binds a [2Fe-2S] cluster and becomes an active repressor to repress the expression of the *sufABCDSE* [7] and optimize the iron-sulfur cluster biogenesis by promoting the expression of the *iscSUA-hscBA-fdx* cluster in *E. coli* cells.

## Summary

**Figure 10.**
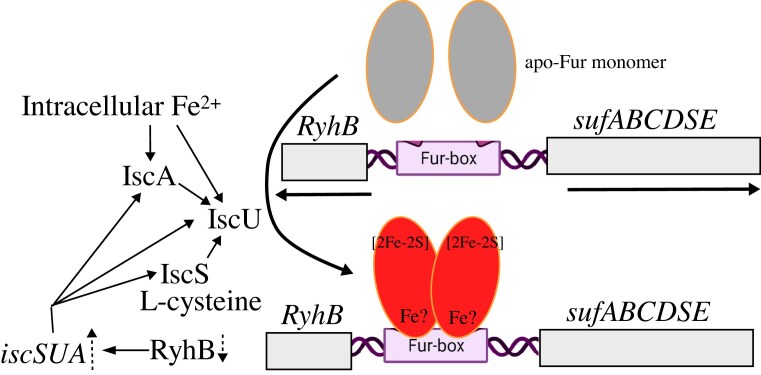
A proposed model for the interplay between iron-sulfur cluster biogenesis and regulation of intracellular iron homeostasis in *E. coli*. When intracellular free iron content is limited, there is no [2Fe-2S] cluster assembled in Fur, and Fur is inactive, resulting in expression of iron import genes, the iron-sulfur cluster assembly gene cluster *sufABCDSE*, and the iron regulatory RNA, RyhB. RyhB promotes degradation of the *iscSUA* mRNA and decreases the house-keeping iron-sulfur cluster assembly activity. When intracellular free iron content is elevated, a [2Fe-2S] is assembled in Fur by the *iscSUA* system, and Fur becomes an active repressor to repress the expression of iron import genes, the gene cluster *sufABCDSE*, and *RyhB*, to optimize intracellular iron content. With the speculation (?), Fur may also bind Fe(II) at site 2 to further enhance its regualtory activity when intracellular free iron content is high.

In *E. coli* and many other bacteria, intracellular free iron content is mobilized and delivered for iron-sulfur cluster biogenesis. Our studies suggested that the iron-sulfur cluster assembly protein IscA may act as an iron chaperone to recruit intracellular free iron content for iron-sulfur cluster biogenesis. When intracellular free iron content is elevated, the global iron regulator Fur assembles a [2Fe-2S] cluster via the iron-sulfur cluster assembly machinery encoded by the housekeeping *iscSUA-hscBA-fdx* cluster and becomes an active pressor to regulate intracellular iron homeostasis. Conversely, the [2Fe-2S]-bound Fur regulates iron-sulfur cluster biogenesis by indirectly promoting the expression of the housekeeping *iscSUA-hscBA-fdx* cluster through repression of the regulatory RNA RyhB and directly repressing the expression of the inducible *sufABCDSE* cluster. When intracellular ‘free’ iron content is scarce, Fur loses the [2Fe-2S] cluster, de-represses the inducible *sufABCDSE* cluster, and inhibits the expression of *iscSUA-hscBA-fdx* cluster to assemble iron-sulfur clusters in proteins in cells without generating excessive oxidative damages (Fig. 10). Thus, Fur represents a key player linking biogenesis of iron-sulfur clusters and regulation of intracellular iron homeostasis in bacteria.

## Data Availability

No new data was generated or analyzed in support of this research.
